# Intrinsically High Capacity of Animal Cells From a Symbiotic Cnidarian to Deal With Pro-Oxidative Conditions

**DOI:** 10.3389/fphys.2022.819111

**Published:** 2022-02-10

**Authors:** Pauline Cotinat, Clara Fricano, Gaëlle Toullec, Eric Röttinger, Stéphanie Barnay-Verdier, Paola Furla

**Affiliations:** ^1^CNRS, INSERM, Institute for Research on Cancer and Aging, Nice, Université Côte d’Azur, Nice, France; ^2^Institut Fédératif de Recherche – Ressources Marines (MARRES), Université Côte d’Azur, Nice, France; ^3^UFR 927, Sorbonne Université, Paris, France

**Keywords:** *Anemonia viridis*, *in vitro* cell cultures, oxidative stress, hydrogen peroxide, cnidarian

## Abstract

The cnidarian-dinoflagellate symbiosis is a mutualistic intracellular association based on the photosynthetic activity of the endosymbiont. This relationship involves significant constraints and requires co-evolution processes, such as an extensive capacity of the holobiont to counteract pro-oxidative conditions induced by hyperoxia generated during photosynthesis. In this study, we analyzed the capacity of *Anemonia viridis* cells to deal with pro-oxidative conditions by *in vivo* and *in vitro* approaches. Whole specimens and animal primary cell cultures were submitted to 200 and 500 μM of H_2_O_2_ during 7 days. Then, we monitored global health parameters (symbiotic state, viability, and cell growth) and stress biomarkers (global antioxidant capacity, oxidative protein damages, and protein ubiquitination). In animal primary cell cultures, the intracellular reactive oxygen species (ROS) levels were also evaluated under H_2_O_2_ treatments. At the whole organism scale, both H_2_O_2_ concentrations didn’t affect the survival and animal tissues exhibited a high resistance to H_2_O_2_ treatments. Moreover, no bleaching has been observed, even at high H_2_O_2_ concentration and after long exposure (7 days). Although, the community has suggested the role of ROS as the cause of bleaching, our results indicating the absence of bleaching under high H_2_O_2_ concentration may exculpate this specific ROS from being involved in the molecular processes inducing bleaching. However, counterintuitively, the symbiont compartment appeared sensitive to an H_2_O_2_ burst as it displayed oxidative protein damages, despite an enhancement of antioxidant capacity. The *in vitro* assays allowed highlighting an intrinsic high capacity of isolated animal cells to deal with pro-oxidative conditions, although we observed differences on tolerance between H_2_O_2_ treatments. The 200 μM H_2_O_2_ concentration appeared to correspond to the tolerance threshold of animal cells. Indeed, no disequilibrium on redox state was observed and only a cell growth decrease was measured. Contrarily, the 500 μM H_2_O_2_ concentration induced a stress state, characterized by a cell viability decrease from 1 day and a drastic cell growth arrest after 7 days leading to an uncomplete recovery after treatment. In conclusion, this study highlights the overall high capacity of cnidarian cells to cope with H_2_O_2_ and opens new perspective to investigate the molecular mechanisms involved in this peculiar resistance.

## Introduction

The evolutionary success of symbiotic cnidarians is based on a mutualism with dinoflagellates of the family Symbiodiniaceae. The symbionts, living inside the gastrodermal host cells, find a protected and stable environment and benefit from inorganic compounds provided by the animal cells (e.g., nitrogen, phosphorus, and sulfate) for their photosynthetic activity. Conversely, the animal host benefits from the organic compounds produced by algal photosynthesis (e.g., glucose and subsequently amino-acids, lipids) and largely transferred from the alga to the animal cell (Davy et al., 2012). This partner cooperation allows autotrophy to the animal host, leading to the colonization of oligotrophic waters by the symbiotic holobiont.

Concomitantly with those advantages, some constraints appear, especially the photosynthetic-dependent production of oxygen in the animal tissue. Such oxygen production causes diurnal hyperoxia condition in a symbiotic cnidarian, leading to a pro-oxidant state with reactive oxygen species (ROS) overproduction (Dykens et al., 1992; Richier et al., 2003; Saragosti et al., 2010; Shaked and Armoza-Zvuloni, 2013). Both partners have the pathways for cross-regulating the intracellular redox state, especially by ROS detoxication through a full suite of antioxidant enzymes to avoid cellular damages (Shick and Dykens, 1985; Richier et al., 2003, 2005; Plantivaux et al., 2004; Furla et al., 2005; Merle et al., 2007; Pey et al., 2017).

The study of ROS sensitivity in these organisms is also of environmental interest. Environmental perturbations (especially variations in temperature and UV radiation) induce oxidative stress that may lead in extreme cases to symbiosis breakdown, a process commonly called bleaching. Thus, under stressful oxidative conditions, Symbiodiniaceae can be eliminated from or exit the host through different cellular processes, like exocytosis, cell detachment, necrosis or apoptosis (see for review Suggett and Smith, 2020). Oxidative stress is known to induce specific cellular damages such as DNA modification (DNA adducts), lipid peroxidation and protein oxidation. In symbiotic cnidarians, several biochemical biomarkers (e.g., protein carbonylation, lipid peroxidation, and protein ubiquitination) were validated in studies following imbalances between ROS overproduction and antioxidant defenses during environmental stress, resulting in the disruption of the symbiotic association (e.g., Lesser and Farrell, 2004; Richier et al., 2006; Pey et al., 2011).

Among ROS, hydrogen peroxide (H_2_O_2_) is a relatively stable chemical formed from O_2_ and is naturally present in the aquatic systems (Ndungu et al., 2019) with concentrations ranging from 0.3 μM in the water column to 4 μM in intertidal areas (Abele-Oeschger et al., 1997). It originates from marine biota (Hansel and Diaz, 2021) or is carried by the rain (Cooper et al., 1987). In all the organisms, intracellular H_2_O_2_ levels can reach tens of micromolar and is generated during normal cellular metabolism (i.e., photosynthesis and respiration) playing crucial roles in the intracellular signaling such as hypoxic signal transduction, cell differentiation and proliferation as well as for immune responses (Halliwell et al., 2000; Apel and Hirt, 2004; Giorgio et al., 2007). At high production levels, the H_2_O_2_ effect can be mitigated by several antioxidant defenses including peroxidases, catalases, thioredoxin reductase, peroxiredoxins, and glutathione S-transferases family enzymes which can lead to rapidly decreasing intracellular H_2_O_2_ concentrations. However, if cellular redox homeostasis cannot be maintained, H_2_O_2_ leads to reversible and irreversible oxidative modifications of proteins (e.g., carbonylation), enhancing protein ubiquitination and subsequent proteasome activation. In addition, cell cycle arrest or apoptosis could also be observed (see for review Fulda et al., 2010). Although the impact of H_2_O_2_ has been more widely investigated in mammalian cells and particularly in tumor cells (see for review Lennicke et al., 2015), studies have shown a similar impact in marine invertebrates, such as bivalves or polychaetes (Abele-Oeschger et al., 1994; McDonagh and Sheehan, 2006, 2007; Da Rosa et al., 2008; Friedman et al., 2018; Nguyen, 2020).

In the coral symbiont, H_2_O_2_ has been shown to be a by-product of photosynthesis processes (Suggett et al., 2008; Armoza-Zvuloni and Shaked, 2014). Thanks to its cell-permeable properties, H_2_O_2_ may diffuse from algal to animal host cells. Interestingly, some studies reported a release of H_2_O_2_ from non-stressed corals (Armoza-Zvuloni and Shaked, 2014) and an extracellular production by the dynamics of the superoxide anion (Saragosti et al., 2010). Therefore, due to their symbiosis lifestyle, animal host cells are daily exposed to H_2_O_2_, raising the question of their intrinsic potential to resist a massive influx of H_2_O_2_. Nevertheless, in excess, ROS (including H_2_O_2_) cause negative impact (mostly on protein and lipids) on the symbiont, leading to photosynthesis impairment, even if no bleaching phenomenon is induced (Higuchi et al., 2009; Roberty et al., 2015, 2016).

*Anemonia viridis* is a temperate sea anemone deeply studied as biological model of the cnidarian-dinoflagellate symbiosis. Its enzymatic antioxidant properties, tissue distribution and regulation have been intensively investigated (Hawkridge et al., 2000; Richier et al., 2003, 2005; Plantivaux et al., 2004; Merle et al., 2007; Ganot et al., 2011; Pey et al., 2017). In addition, the sensitivity of *A. viridis* to thermal and UV stresses has been well described and some mechanisms of bleaching have been decrypted, including oxidative stress and apoptosis (Richier et al., 2006; Moya et al., 2012). Recently, we succeeded in the establishment of primary cell cultures from *A. viridis* exhibiting a gastrodermal signature (Barnay-Verdier et al., 2013; Ventura et al., 2018; Fricano et al., 2020). Thus, to test the hypothesis of an intrinsic resistance of animal cells to H_2_O_2_, we exposed *A. viridis* specimens and primary cell cultures at the same H_2_O_2_ concentrations (200 and 500 μM) during the same periods of time (24 h and 7 days), and compared the respective responses. For each treatment, we monitored global health parameters (symbiotic state, viability and cell growth) and stress biomarkers (global antioxidant capacity, oxidative protein damages, and protein ubiquitination). This allowed us to assess the cnidarian cell susceptibility to H_2_O_2_ exposure, highlighting the putative influence of the tissue organization or/and of the presence of symbionts.

## Materials and Methods

### Biological Material

#### *Anemonia viridis* Specimens

Specimens of *A. viridis* (Forskal 1775) were collected (prefectural authorization n107; 28 February 2019) from “Plage des ondes,” Antibes, France, (43°33′17″ N, 7°07′17.7″ E), and maintained in a closed-circuit aquarium with artificial seawater (ASW, Prodibio Expert Reef Salt) at 36–38‰ at 18.0 ± 0.5°C with weekly water changes. A LED bar (450 nm—Deckey LED aquarium) provided light at a constant saturating irradiance of 100 μmol m^–2^s^–1^ (measured using a special sensor QSL-100, Biospherical Instruments Inc., San Diego, CA, United States) on a 12 h:12 h (light:dark) photoperiod. Sea anemones were fed once a week with oysters.

#### Gastrodermal Primary Cell Cultures

Independent primary cell cultures were obtained from different *A. viridis* individuals and maintained as described in Ventura et al. (2018) and Fricano et al. (2020). Briefly, cells were cultured in the dark in a thermo-regulated incubator (POL-EKO-APARATURA, Poland) at 20.0 ± 0.5°C. The optimized culture medium was replaced weekly and consisted of 20% GMIM (Gibco, Carlsbad, CA, United States), 5% fetal bovine serum (FBS; PAA/GE Healthcare, Chicago, IL, United States), 1% kanamycin (100 μg/mL, Sigma-Aldrich), 1% amphotericin B (2.5 μg/mL; Interchim, Montluçon, France), 1% antibiotic antimycotic solution (Sigma-Aldrich), 1% L-glutamate (Sigma-Aldrich), and 71% of filtered ASW. The medium was adapted in respect to the Mediterranean Seawater characteristics (i.e., salinity 40 ppt and pH 8.1).

### Hydrogen Peroxide Experimental Design

#### *In vivo* Experiments

Eight specimens of *A. viridis* were kept individually in 5 L tanks under controlled conditions. Six tentacles were cut from each specimen after 24 h and 7 days to assess the control condition. For 200 and 500 μM H_2_O_2_ treatments, a solution of H_2_O_2_ (Sigma-Aldrich) was added in each tank (200 μM for four of them and 500 μM for the four others). After 24 h and 7 days of treatment, six tentacles were cut from each specimen to assess the treatment condition. All tentacles from the different time points and conditions were longitudinally opened and the gastroderm (containing the dinoflagellates) was manually separated from the epiderm. A centrifugation at 1,000 × *g* for 5 min was used to separate the gastrodermal compartment from the dinoflagellates fraction (Richier et al., 2003).

#### *In vitro* Experiments

The cell response to H_2_O_2_ treatment was assessed on at least three independent primary cell cultures. Cells seeded in 12-well plates were exposed to H_2_O_2_ treatment (Sigma-Aldrich) starting with a dose-response experiment (between 0 and 2 mM) for 7 days. Another set of experiments was performed by incubating cells to 0 (control), 200 and 500 μM H_2_O_2_ for short term (1, 2, and 6 h) or long term (1 day or 7 days) exposure. We then conducted resilience experiments by reseeding the treated cells in their normal culture medium for 7 days to assess their recovery capacity. Three wells, for each condition and time point, were used for all analyses.

### Symbiotic State Assessment of *A. viridis* Specimens

The density of the endosymbionts from the genus *Philozoon* sp. (LaJeunesse et al., 2021) was assessed according to Zamoum and Furla (2012). Briefly, two tentacles were cut from each animal before and after H_2_O_2_ treatment in each condition (0, 200, or 500 μM H_2_O_2_) and then put in 2 M solution of NaOH and incubated at 37°C for 1 h to dissolve all animal tissue. To determine symbiont density, three replicate samples were counted using a modified Neubauer hemocytometer (Sigma-Aldrich). The remaining extract was used to determine the protein content from which we normalized symbiont density.

### Protein Extractions

Gastrodermal cells from primary cultures and the animal’s epidermal and gastrodermal tissues were all separately placed in 200 μL of lysis extraction buffer (HEPES 25 mM, MgCl2 5 mM, EDTA 5 mM, DTT 5 mM, and PMSF 2 mM) and homogenized. Tissue samples were potterized, and both tissue and cell samples were sonicated and centrifuged to obtain the cytosoluble protein content. The total protein concentration of each sample was then assessed by a Bradford assay (Bradford reagent; SIGMA-ALDRICH), using 0–2 μg/ml bovine serum albumin solutions as standard curve.

### Cell Viability and Growth Rate of Cultivated Cells

Cell viability was assessed by evaluating the membrane integrity using the Evans blue method. Therefore, viable cells (unstained) and dead cells (stained) were identified and counted on a Neubauer improved hemocytometer (Sigma-Aldrich) using an optic microscope (Zeiss Axio Imager Z1). The cell viability was defined as the percentage of viable cells relative to total cells (i.e., viable and dead cells). Cell growth rate was assessed every week using the cells counts with Evans blue method, as previously described in Ventura et al. (2018) and Fricano et al. (2020). The following formula was then used to calculate the weekly growth rate:


$$\mathrm{Growth}\,\mathrm{rate}=\frac{(\mathrm{Viable}\mathrm{cells}\left(d+7\right)-\mathrm{Viable}\mathrm{cells}\left(d\right)}{\mathrm{Viable}\,\mathrm{cells}\,\left(d\right)}\,d=\mathrm{day}$$

### Total Oxidative Scavenging Capacity Assay

The oxygen radical scavenging activities of protein samples were determined using fluorometric assay according to Naguib (2000). Protein samples are incubated with phosphate buffer (75 mM pH 7.5), in presence of 180 nM 6-carboxyfluorescein as fluorescent probe and 36 mM AAPH [2,2′-azobis(2-amidinopropane) dihydrochloride] as the peroxyl radical generator. In the assay, Trolox [6-hydroxy-2,5,7,8-tetramethylchroman-2-carboxylic acid] is used as antioxidant calibrator. Fluorescence recordings were performed in black microplates (96-wells Greiner Bio-One), and fluorescence decay was measured by spectrofluorometer (Safas, Monaco) every minute for a total duration of 1 h at an excitation/emission wavelength of 520/495 nm. Relative antioxidant activities of protein samples (tested in duplicate) were measured by comparison with Trolox standard. Results were expressed in Trolox equivalents and represented in the figures as a ratio to the control condition.

### Protein Carbonylation Analysis

Carbonyl content of the cytosolic extractions was measured using an ELISA assay and spectrophotometry, according to Buss et al. (1997). Protein derivatization was done by adding a dinitrophenylhydrazine (DNP) solution (10 mM in 6 M guanidine hydrochloride, 0.5 M potassium phosphate buffer) to the protein samples. The ELISA assay used anti-DNP antibody produced in rabbit (1:500; Sigma-Aldrich) and anti-rabbit Ig (1:2000; Bio-Rad). 0–100% reduced bovine serum albumin (BSA) were used as standard curve. Carbonyl content of protein samples was expressed in nanomoles per milligram of protein and was then represented in the figures as a ratio to the control condition.

### Ubiquitin Conjugates Dot-Blot Analysis

Ubiquitinated proteins were assayed, according to Haas and Bright (1985), by dot-blotting 3 μg of protein samples fixed to a nitrocellulose membrane, which was incubated with primary antibody (1:1000; Mono- and polyubiquitinylated conjugates recombinant monoclonal antibody; Enzo Life Sciences). The membrane was next incubated with the secondary antibody (1:5000; anti-mouse antibody; Sigma-Aldrich). After chemiluminescence revelation (ECL), levels of spot density were measured using image analysis on GeneTools (SynGene). Levels of ubiquitinated proteins were obtained after normalization by spot density measured after amido black solution staining. Results were represented in the figures as a ratio to the control condition.

### Detection of Intracellular Reactive Oxygen Species

Intracellular ROS were detected in cultivated cells treated or not with H_2_O_2_ using a sensitive fluorescent probe that penetrates the cell and, when oxidized by intracellular free radicals, binds to DNA, emitting a more intense green fluorescence (CellROX™ Green Reagent, Invitrogen). We followed the manufacturer’s instructions. Briefly, 10 μL of a 500 μM solution of CellROX™ Green were added to each treated well during 30 min at room temperature in the dark. After rinsing, cells were resuspended in 200 μL of PBS 0.6 M. Samples were then analyzed by flow cytometry (CytoFLEX LX, Beckman Coulter), excited at 488 nm and detected at 515–530 nm. For data analysis, we selected the green-fluorescent cells (CellROX-positive cells) within the population of interest. The population of interest corresponds to the cell population from which the analysis has been conducted after hierarchical exclusion of debris and doublets. For each experiment, controls (i.e., untreated cells) allowed to evaluate the basal intracellular ROS level in the cell cultures.

### Statistical Analysis

For all cellular and biochemical markers evaluated in this study, the effect of the different treatments was analyzed with a two-way ANOVA test or Kruskal–Wallis test followed by, respectively, a Tukey’s or Dunn *post hoc*, depending on the homoscedasticity of the data set. All experiments, i.e., *in vivo* and *in vitro*, were conducted on at least three independent biological replicates.

## Results

### Hydrogen Peroxide Effects on *A. viridis* Specimens

**FIGURE 1:**
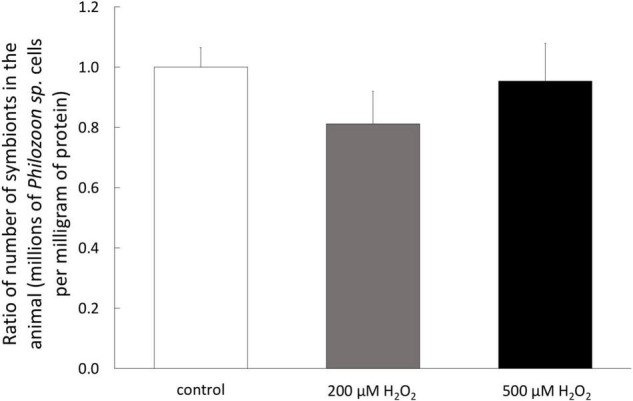
Symbiotic state of *A. viridis* specimens under H_2_O_2_ treatments. Content of *Philozoon* sp. symbionts in the tentacles of *A. viridis* before and after 7 days of 0 μM (control, white bars) 200 μM (gray bars) and 500 μM (black bars). At day 0, the *A. viridis* specimens hosted 2.3 × 10^6^ symbiont mg^–1^. Data are represented as means with standard error bars.

To determine the susceptibility of *A. viridis* specimen to H_2_O_2_, we monitored the sign of bleaching, tissue necrosis and potential mortality during 7 days of exposure. No sign of tissue necrosis or mortality of the *A. viridis* specimens have been observed during H_2_O_2_ exposure. In addition, no differences in the symbiotic state have been measured since the quantity of symbionts, *Philozoon* sp. genus, per mg of protein remained the same after 7 days of treatment with both H_2_O_2_ concentrations (Figure 1). In addition, the sea anemones were also monitored several weeks after the treatment, and no signs of bleaching or disease were observed (data not shown).

### Hydrogen Peroxide Effects on Stress Biomarkers of *A. viridis* Tissues Under Treatments

**FIGURE 2:**
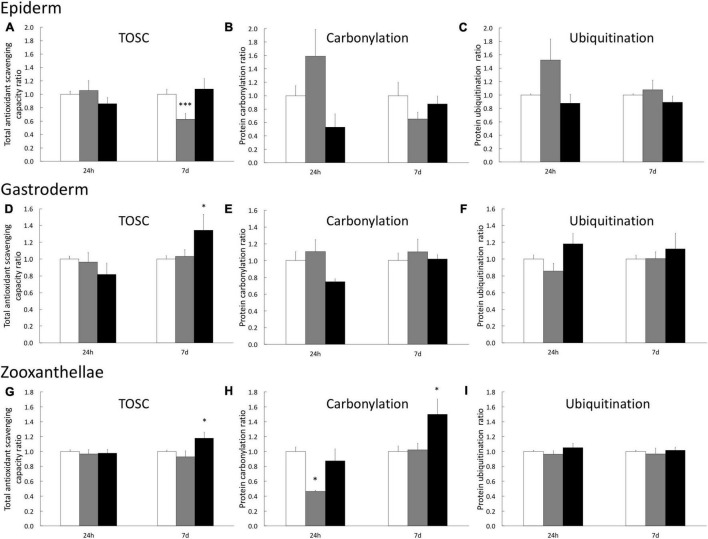
Stress biomarkers in *A. viridis* tissues under H_2_O_2_ treatments. Measurements have been performed separately on animal’s epiderm extracts **(A–C)**, on animal’s gastroderm extract **(D–F)** and on symbiont extracts **(G–I)** from the gastroderm. Total Oxidative Scavenging Capacity **(A,D,G)**, protein carbonylation **(B,E,H)** and protein ubiquitination **(C,F,I)** were measured under 0 μM H_2_O_2_ (control; white bars), 200 μM H_2_O_2_ (gray bars) and 500 μM H_2_O_2_ (black bars). Data are expressed relative to the control condition (ratio of the control condition). Means values with standard error bars are presented. Significant differences between control and stress condition are expressed by *p < 0.05 and ^***^p < 0.001.

To analyze the impact of H_2_O_2_ at the tissue level, we assessed three biochemical stress markers (TOSC, carbonylation, and ubiquitination) known to be associated with oxidative stress conditions, in the three tissue compartments (epiderm, gastroderm, and symbiont) separately. In the epiderm, we only observed a slight but significant decrease of TOSC after 7 days of 200 μM H_2_O_2_ (Figures 2A–C). It is noteworthy that after 1 day of 200 μM H_2_O_2_, we observed a trend of increasing protein damages (carbonylation and ubiquitination), which totally disappeared after 7 days.

In the gastrodermal compartment (i.e., animal tissue), we only observed an effect of H_2_O_2_ treatments after 7 days of 500 μM H_2_O_2_, inducing a 34% increase of TOSC (Figure 2D) which is sufficient to avoid protein damages (Figures 2E,F). Finally, the analysis on the symbiont fraction showed an increase of TOSC (17% higher than the control), associated with an increase of protein carbonylation level (50% higher than the control) after 7 days at 500 μM H_2_O_2_ (Figures 2G,H). No impact on symbiont protein ubiquitination has been measured (Figure 2I). Taken together these data suggest a higher capacity of cnidarian animal cells to cope with H_2_O_2_ and prevent protein damages compared to the algal symbiont.

### Dose Response of Gastrodermal Cultivated Cells to Hydrogen Peroxide

**FIGURE 3:**
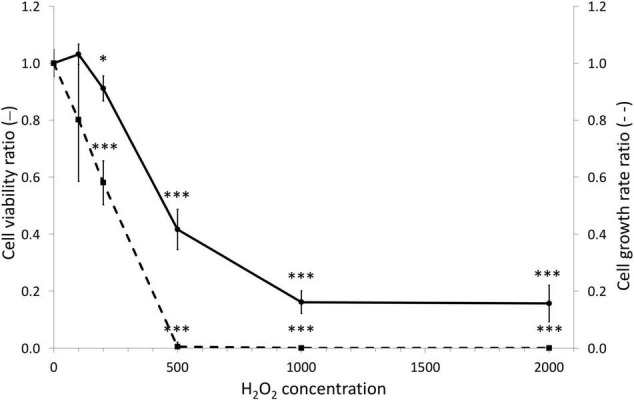
Dose viability and growth responses of gastrodermal cultivated cells under H_2_O_2_ treatments. *A. viridis* gastrodermal cultivated cells were treated with H_2_O_2_ at different concentrations for 7 days before measuring cell viability (solid line) and cell growth rate (dashed line). Cell viability and growth rate are expressed relative to the control condition (ratio of the control condition). Means values with standard error bars are presented. Significant differences between control and stress condition are expressed by *p < 0.05 and ^***^p < 0.001.

To gain more insight in the capacity of the animal gastrodermal compartment to cope with ROS we first assessed the overall toxicity of H_2_O_2_ using gastrodermal cell cultures from *A. viridis*. More precisely, we applied a range of H_2_O_2_ concentrations from 100 μM to 2 mM for 7 days and assessed cellular growth and viability (Figure 3). Without any treatment, the mean viability of cultivated cells was 93% ± 1.17 and the weekly growth rate was 18.7 ± 4.5. At 100 μM, no effect on viability and growth rate was observed. The first significant effects on both parameters appeared at 200 μM (10% viability and 42% growth rate decreases) and drastically exacerbated at 500 μM. With a concentration of 500 μM H_2_O_2_, the viability was decreased by 58% (EC_50_ = 400.8 μM) and the growth was totally arrested. These results led us to direct further experiments toward the concentrations of 200 and 500 μM H_2_O_2_, where the response of the cells seemed pivotal and allowing the comparison with the *in vivo* experiments.

### Hydrogen Peroxide Effects on Oxidative Status of Gastrodermal Cultivated Cells

**FIGURE 4:**
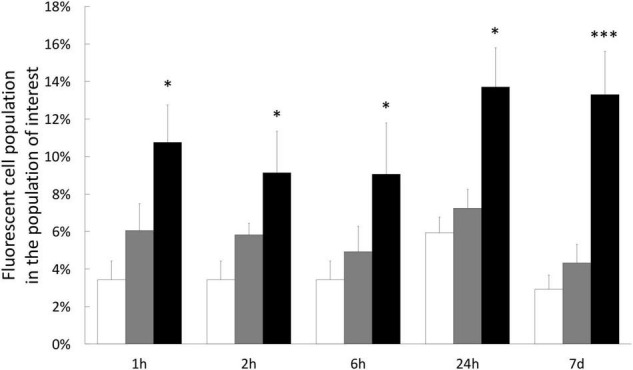
Quantification of intracellular ROS in gastrodermal cultivated cells under H_2_O_2_ treatments. Intracellular content of ROS was measured by staining cells with CellROX Green Reagent after 1 h, 2 h, 6 h, 1 day, or 7 days with 0 μM H_2_O_2_ (control condition; white bars), 200 μM H_2_O_2_ (gray bars) or 500 μM H_2_O_2_ (black bars). Results represent the percentage of fluorescent cells (CellROX-positive cells), within the population of interest, quantified by flow cytometry. Data are represented as means with standard error bars. Significant differences between control and treatments are expressed by *p < 0.05 and ^***^p < 0.001.

To test whether H_2_O_2_ treatments induced an intracellular ROS increase, we stained cultivated cells with specific fluorescent probe (CellROX™ Green) allowing to quantify the positive cells for CellROX™ Green. The level of intracellular ROS was thus correlated to the percentage of positive cells for CellROX™ Green. Interestingly, this analysis revealed that although an increasing trend, there was no significant difference between the control (no treatment) and the cells treated with 200 μM H_2_O_2_, whatever the time point (Figure 4). However, there was a significant increase of ROS-positive cells (2.7 times higher in average, 11.2% vs. 4.1%) within the cells treated with 500 μM H_2_O_2_ from 1 h until 7 days of treatment.

### Hydrogen Peroxide Effects on Cellular Parameters Under Treatments and Resilience Phase

**FIGURE 5:**
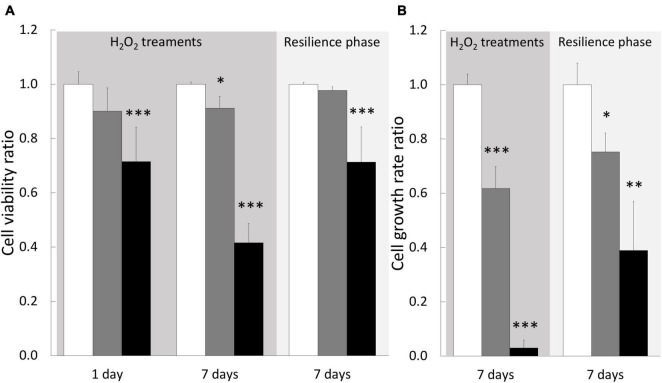
Cellular parameters of gastrodermal cultivated cells under H_2_O_2_ treatments and resilience phase. Cell viability **(A)** and cell growth rate **(B)** were measured under cell treatment with H_2_O_2_ at 200 and 500 μM and during resilience phase (back to control condition for 7 days): 0 μM H_2_O_2_ (control condition; white bars), 200 μM H_2_O_2_ (gray bars) and 500 μM H_2_O_2_ (black bars). Cell viability and growth rate are expressed relative to the control condition (ratio of the control condition). Data are represented as means with standard error bars. Significant differences between control and treatments are expressed by *p < 0.05, ^**^p < 0.01, and ^***^p < 0.001.

**FIGURE 6:**
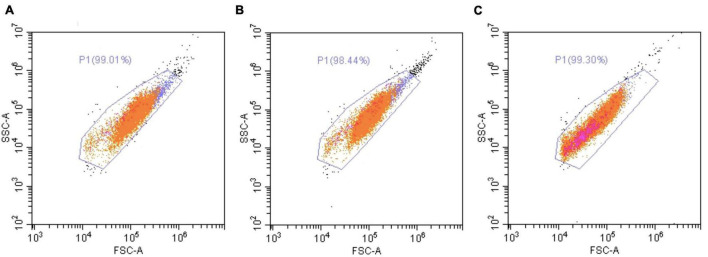
Flow cytometry analysis of *A. viridis* cultivated cells under a 7-days treatment with H_2_O_2_. Flow cytometry analysis were performed on cultivated cell population by excitation at 488 nm and detection at 515–530 nm, after 7 days of incubation without H_2_O_2_ **(A)** or with 200 μM **(B)** or 500 μM **(C)** of H_2_O_2_. Representative dot plots of all experiments performed are shown. Orange dots: cells of interest; pink dots: green-autofluorescent cells.

We next wondered whether H_2_O_2_ exposures induce a stress at the cellular level by monitoring cellular parameters during treatments. In addition, we also evaluated the resilience capacity of treated cells by following the same parameters after a week-recovery phase. Firstly, the analysis of the cell viability, during 1–6 h of exposures, showed no impact of 200 or 500 μM H_2_O_2_ treatments (see Supplementary Figure 1). At 200 μM, there was only a light significant decrease in cell viability (9%) after 7 days of exposure, which was totally recovered after the resilience period (Figure 5A). At 500 μM, a 28% decrease was observed at 1 day that worsened after 7 days (58% of decrease, Figure 5A), confirming our results obtained during the dose-response assay. After a resilience period, only a partial recovery was observed (71% of the control condition), suggesting that not all the cells that survived may be able to compensate the H_2_O_2_–induced stress. In line with this hypothesis, the analysis of the cell population by flow-cytometry showed, after 7 days of 500 μM H_2_O_2_, a modification of cell-population pattern associated with a significant increase of autofluorescence (in average: 3.86% vs. 0.53% for the control condition; *p*-value = 0.021; Figure 6), which could be reflecting cell apoptosis.

The growth rate measurements showed a significant impact of both treatments, 200 and 500 μM H_2_O_2_, with a dose-dependent response as we observed a decrease of 38 and 97%, respectively (Figure 5B). This H_2_O_2_ dose-dependent response was maintained after the resilience period with a partial growth rate recovery for both treatments (75 and 39% of control, respectively, for 200 and 500 μM H_2_O_2_). Taken together these data suggested that 200 μM H_2_O_2_ can be considered as the tolerance threshold of cultivated cnidarian cells while 500 μM H_2_O_2_ can represent a critical concentration inducing deleterious cellular injuries.

### Hydrogen Peroxide Effects on Stress Biomarkers Under Treatments and Resilience Phase

**FIGURE 7:**
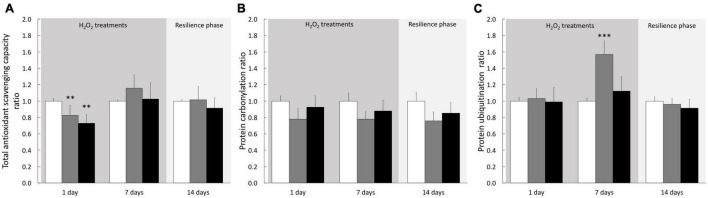
Stress biomarkers in *A. viridis* cultivated cells under H_2_O_2_ treatments and resilience phase. Total Oxidative Scavenging Capacity, TOSC **(A)**, protein carbonylation **(B)** and protein ubiquitination **(C)** were measured under cell treatment with H_2_O_2_ at 200 and 500 μM and during resilience phase (back to control condition for 7 days): 0 μM H_2_O_2_ (control; white bars), 200 μM H_2_O_2_ (gray bars) and 500 μM H_2_O_2_ (black bars). Data are expressed relative to control condition (ratio of the control condition). Means values with standard error bars are presented. Significant differences between control and treatments are expressed by ^**^p < 0.01 and ^***^p < 0.001.

To determine the cellular response induced by H_2_O_2_ treatments on cultivated cells we analyzed biochemical stress markers (TOSC, carbonylation, and ubiquitination). After 1–6 h of treatment, we observed no significant effect on any of the three biomarkers (Supplementary Figure 2). However, after 24 h of treatment TOSC was impaired at both 200 and 500 μM H_2_O_2_. A full recovery was observed after 7 days of treatment and maintained during the resilience phase (Figure 7A), suggesting an acclimation of the cells to the pro-oxidant conditions. No signs of protein damage (protein carbonylation and ubiquitination) were measured after 24 h. However, the 200 μM H_2_O_2_ treatment induced an increase of ubiquitinated proteins after 7 days (Figure 7C). This effect was totally abolished after the resilience period (Figure 7C). These data suggest that H_2_O_2_ exposures, even at 500 μM and after 7 days, didn’t lead to persistent oxidative damages on proteins in cnidarian cultivated cells.

## Discussion

### Extended Tolerance of Symbiotic Cnidarians to Hydrogen Peroxide

In this study, we used H_2_O_2_ to induce pro-oxidative condition and to investigate the stress response in the symbiotic sea anemone *A. viridis* at both whole animal and cellular scales. The H_2_O_2_ concentrations used in the present study correspond to extremely high levels never measured in the seawater. However, rainwater can temporally induced increase of H_2_O_2_ to tens of micromolar (Ndungu et al., 2019; Jones and Lee, 2020). Benthic marine organisms from coastal areas are therefore regularly facing H_2_O_2_, that could lead to oxidative stress (Abele-Oeschger et al., 1997). Indeed, among ROS, H_2_O_2_ is the most abundant and long-lived in sea water and contrary to other ROS, H_2_O_2_ could rapidly diffuse across membranes (see for review Halliwell and Gutteridge, 2015).

In our study model, at both whole animal and cellular scales, 200 μM of H_2_O_2_ did not create a condition of stress, since very weak impact was observed in global health: no sign of mortality or bleaching on specimens (Figure 1), no intracellular ROS accumulation (Figure 4) and only very slight effect on viability after 7 days exposure in cell cultures (Figure 5). In addition, no effect was observed on any stress biomarkers we tested (Figures 2, 7). In cell cultures, we nevertheless measured a significant cellular growth arrest (Figure 5), reflecting a common feature of stress response and could be interpreted as an usual resistance mechanism (see for review Davies, 1999, 2000).

H_2_O_2_ usually represents a threat for most organisms. Indeed, in diverse mammalian cell lines, a cytotoxic effect of H_2_O_2_ could be observed from 60 μM (Coyle et al., 2006) with drastic and irreversible impacts (i.e., apoptosis and necrosis) induced at 400 μM H_2_O_2_ (Xiang et al., 2016). Some studies performed on marine invertebrates showed that micromolar concentrations of H_2_O_2_, ranging from 0.5 to 20 μM, could impact the metabolism of the whole animal. For example, a 40% drop in O_2_ consumption was observed in the Polychaete *Nereis diversicolor* under 5 μM of H_2_O_2_ (Abele-Oeschger et al., 1994), while higher level of H_2_O_2_ (50 μM) can cause oxidative damages (i.e., lipid peroxidation), as observed in another Polychaete species, *Laenoereis acuta* (Da Rosa et al., 2008).

Contrasting with those results, our study highlighted an extended tolerance of symbiotic cnidarian facing even greater H_2_O_2_ concentrations. This resistance to H_2_O_2_ is, however, not a general cnidarian feature as it has been shown that concentrations exceeding 163 μM H_2_O_2_ caused mortality of the non-symbiotic sea anemone *Nematostella vectensis* (Friedman et al., 2018). Therefore, these results reinforced the hypothesis of the adaptation of symbiotic cnidarians to pro-oxidative conditions, due to their lifestyle with a photosynthetic symbiont. Indeed, it has been already highlighted that symbiotic cnidarians exhibited a wide diversity of biochemical antioxidant actors, compared to non-symbiotic species (Furla et al., 2005). For instance, higher number of superoxide dismutase (SOD) isoforms was identified in the symbiotic cnidarian *A. viridis* compared to the non-symbiotic one *Actinia schmidti*. In complement, another comparative analysis showed that the glutathione peroxidase (GPx) isoforms were less numerous in the non-symbiotic sea anemone *N. vectensis* than in the symbiotic sea anemone *A. viridis* (Pey et al., 2017). To confirm the hypothesis of the adaptative process, it will be required to extend the comparison of the antioxidant battalion between symbiotic and non-symbiotic cnidarians at multiple scales, even by including the non-enzymatic actors.

### Importance of Host Cells to Hydrogen Peroxide Defense

In the present study, H_2_O_2_ exposure on whole organism affected mainly the endosymbiont, *Philozoon* sp. genus (LaJeunesse et al., 2021), rather than the animal host tissues. Although antioxidant defenses were stimulated after 7 days, an increase of protein carbonylation was measured in the symbiont fraction, whereas no increase was observed in the animal compartments (Figure 2). The susceptibility of free-living algae to H_2_O_2_ is well documented and highlighted an important heterogeneity in H_2_O_2_ response. For example, although the cyanobacterium *Synechococcus aeruginosus* tolerated until 2 mM H_2_O_2_, another cyanobacterium species, *Microcystis aeruginosa*, was affected by around 20 μM (EC50) and the diatoms *Navicula seminulum* by 200 μM (Drábková et al., 2007). Few studies have been performed on Symbiodiniaceae sensitivity to ROS and again they highlighted species-specific impacts on photosynthesis (Wietheger et al., 2015; Roberty et al., 2016). For example, cultured *Symbiodinium microadriaticum* strain showed high resistance to 1 mM H_2_O_2_, compared to *Fugacium kawagutii* showing drastic damage to photosystem function at the same H_2_O_2_ concentration (Wietheger et al., 2015). In addition, light exposure increased the photosynthesis impairment of cultured Symbiodiniaceae from 30 min of >1 mM H_2_O_2_ exposure (Wietheger et al., 2015).

Protected inside the gastrodermal host cell, the endosymbiont would not have shown any signs of oxidative stress under H_2_O_2_ exposure, but the sensitivity assessed in our study did not confirm this assumption. This sensitivity to H_2_O_2_ could, however, be correlated to previous studies demonstrating that Symbiodiniaceae living *in hospite* present a reduction of the antioxidant enzymatic defenses (i.e., SOD, catalases or peroxidases) compared to the free-living condition (Lesser and Shick, 1989; Richier et al., 2005; Pey et al., 2017). Nevertheless, it has been shown that *in hospite*, Symbiodiniaceae harbor a higher surface volume of thylakoid lamellae (Lesser and Shick, 1990), increasing definitely the photosystem density and consequently the source of ROS associated with electron chain transports (Saragosti et al., 2010).

These data, in addition with measurements of H_2_O_2_ diffusion from the symbiont (Suggett et al., 2008; Armoza-Zvuloni and Shaked, 2014), support the conclusion that, in *hospite*, the redox homeostasis of the symbiont is bolstered by the antioxidant defenses of the animal host cells. For example, in *A. viridis* it has been frequently observed that the animal compartment constitutes the major contributor to the holobiont antioxidant potential, with higher amount of antioxidant defenses compared to the symbiont fraction (Richier et al., 2003, 2005; Plantivaux et al., 2004; Merle et al., 2007; Pey et al., 2017). This agrees with studies performed on other symbiotic cnidarian species (Yakovleva et al., 2004; Levy et al., 2006; Krueger et al., 2015). By consequence, an experimental burst of H_2_O_2_ leads to more deleterious effects in the endosymbiont than in animal cells, illustrating their capacity to cope with H_2_O_2_.

### No Bleaching Induction by Hydrogen Peroxide

Even at highest H_2_O_2_ exposure, no bleaching was observed neither during, nor after the exposure period in the treated *A. viridis* specimens (Figure 1). Interestingly, despite the protein damages observed in the symbiont, the equilibrium of the symbiosis was maintained. It has been largely documented in symbiotic cnidarians that stress-induced bleaching (e.g., thermal stress) is linked with the over-production of ROS by the endosymbiont, leading to significant oxidative damages in the host cells (see for review Suggett and Smith, 2020). Due to its permeable properties and its overproduction in several strains of Symbiodiniaceae exposed to thermal stress (Lesser, 1996; Suggett et al., 2008; Roberty et al., 2015), this ROS has then been suggested to be responsible of oxidative stress occurring in host cells during bleaching events. However, in this study, the absence of bleaching and of oxidative damages in host cells under H_2_O_2_ exposures can support the conclusion that H_2_O_2_ may not be the most important ROS associated with coral bleaching.

### Limits of the Resistance

At cellular level, pro-oxidative condition can elicit a broad spectrum of responses from proliferation to growth arrest, or senescence and cell death, depending on the cell capacity to overcome the stress by repairing or removing damaged molecules. The observed effect reflects the balance between intracellular pathways activated in response to the oxidative injury and can vary significantly with the concentration of the oxidant agent and the treatment exposure. In our *in vitro* study model, 500 μM of H_2_O_2_ induced a decrease of cell viability, particularly pronounced after 7 days of treatment and associated with a drastic growth arrest (Figure 5).

This response could be explained by a strategy of the “sacrifice” signaling pathway set up by the cells to eliminate the most damaged cell population (Davies, 2000). Indeed, a change in the cell population pattern was highlighted after 7 days at 500 μM H_2_O_2_ (Figure 6) and no protein damages were observed in these surviving cells (Figure 7). Nevertheless, the increase of autofluorescence measured in surviving cells after 7 days at 500 μM H_2_O_2_ (Figure 6) might be correlated with changes in metabolic activity of mitochondria that cells undergo during apoptosis, as it has been previously observed in mammalian cells (Levitt et al., 2006). In addition, the partial recovery of cell growth after the resilience period (Figure 5) suggested a non-reversible impact of 500 μM H_2_O_2_ injury on surviving cells, whose mechanisms should be deeply addressed in the future.

Finally, comparing the *in vitro* and the *in vivo* approaches, we highlighted that the sensitivity of *A. viridis* gastrodermal cells at 500 μM of H_2_O_2_ exposure is less pronounced in the gastrodermal tissue than in the isolated cultivated cells (Figures 2, 7). This is likely due to the contribution of the tissue organization thanks to host cell/cell communication, more efficient turnover of damaged cells and/or by a signaling pathway linked to the presence of the symbiont.

Moving forward thanks to the *in vitro* cnidarian cell culture, an ambitious perspective of this study will be to disentangle the mechanisms of H_2_O_2_ resistance of cnidarian cells and more specifically to assess the impact of other ROS (as the superoxide anion and the hydroxyl radical), thus contributing to decipher the adaptative tools that have evolved for a successful symbiosis stability and conversely to better understand the bleaching processes.

## Data Availability Statement

The original contributions presented in the study are included in the article/Supplementary Material, further inquiries can be directed to the corresponding author/s.

## Author Contributions

PC did all the investigations (experimental work) and statistical analyses. CF contributed to the obtaining of cell cultures and their maintenance. CF and GT initialized the H_2_O_2_ experiments on cell cultures. PF and SB-V designed and supervised the research. PC, ER, SB-V, and PF wrote the manuscript. All authors read and agreed to the published version of the manuscript.

## Conflict of Interest

The authors declare that the research was conducted in the absence of any commercial or financial relationships that could be construed as a potential conflict of interest.

## Publisher’s Note

All claims expressed in this article are solely those of the authors and do not necessarily represent those of their affiliated organizations, or those of the publisher, the editors and the reviewers. Any product that may be evaluated in this article, or claim that may be made by its manufacturer, is not guaranteed or endorsed by the publisher.
