# Supplementary material for: Intrinsically High Capacity of Animal Cells From a Symbiotic Cnidarian to Deal With Pro-Oxidative Conditions
Source: Front Physiol. 2022 Feb 10;13:819111. doi: 10.3389/fphys.2022.819111 (PMC8867213; doi:10.3389/fphys.2022.819111)
Supplement: Supplementary file 1 [file Data_Sheet_1.docx]

**Supplementary file**

**
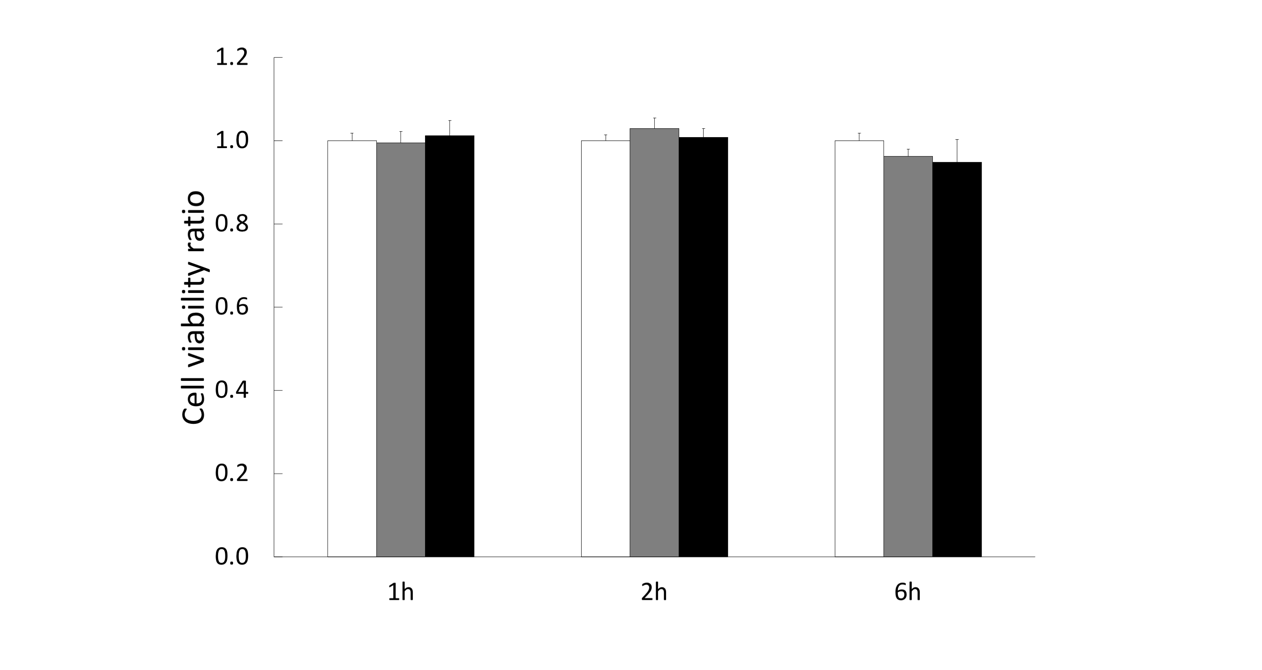
**

**Figure S1: Cell viability of *A. viridis* cultivated cells under 1, 2 or 6 hours H_2_O_2_ treatment.** Cell viability was measured under cell treatment with H_2_O_2_ at 200 and 500 µM and during 1, 2 or 6 hours: 0 µM H_2_O_2_ (control condition; white bars), 200 µM H_2_O_2_ (grey bars) and 500 µM H_2_O_2_ (black bars). Data are represented as means with standard error bars. No significant difference was observed.

**
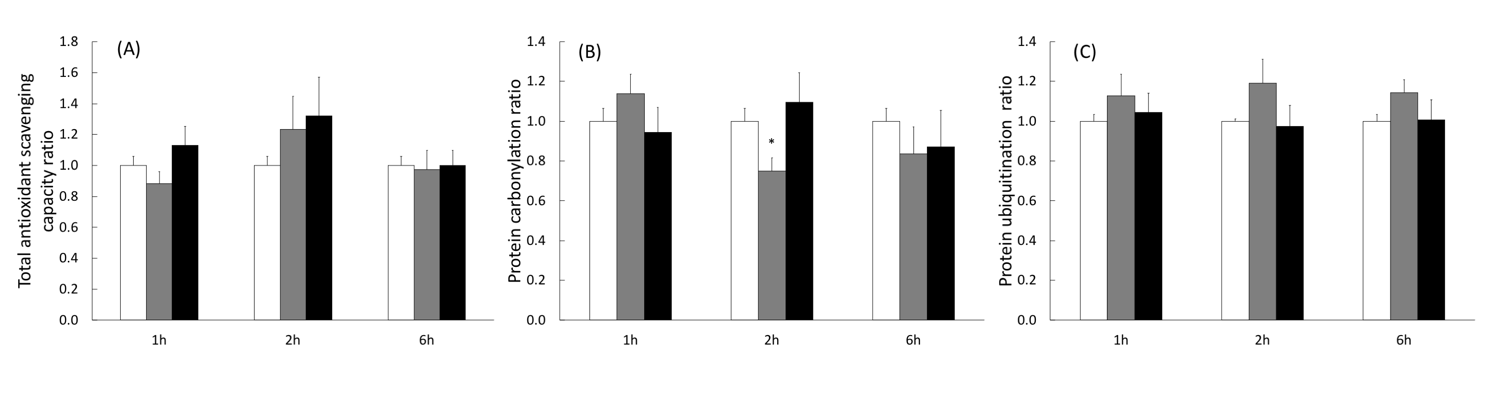
**

**Figure S2: Stress biomarkers in *A. viridis* cultivated cells under 1, 2 or 6 hours H_2_O_2_ treatments.** Total Oxidative Scavenging Capacity, TOSC (A), protein carbonylation (B) and protein ubiquitination (C) were measured under cell treatment with H_2_O_2_ at 200 and 500 µM during 1, 2 or 6 hours : 0 µM H_2_O_2_ (control; white bars), 200 µM H_2_O_2_ (grey bars) and 500 µM H_2_O_2_ (black bars). Data are represented as means with standard error bars. Significant difference between control and treatments is expressed by * (p<0.05).
